# Tourniquet use in patients with sickle cell trait (SCT): Mediterranean or African ancestry influences complications, demonstrating a higher prevalence than control patients: matched study of nine hundred and forty SCT versus one thousand, two hundred and sixty three non-SCT patients

**DOI:** 10.1007/s00264-025-06555-8

**Published:** 2025-05-07

**Authors:** Philippe Hernigou, Paul Vedrenne, Sami Karam, Charles Henri Flouzat-Lachaniette

**Affiliations:** Paris-Est Créteil University, Créteil, France

**Keywords:** Sickle cell trait, Tourniquet, Orthopaedic surgery, Thrombophlebitis, Diabetes, Pulmonary embolism

## Abstract

**Purpose:**

One ongoing debate in orthopaedic surgery concerns using tourniquets in sickle cell trait (SCT) patients. SCT, a heterozygous carrier state of sickle cell disease (SCD), affects an estimated 300 million individuals globally with various genetic ancestries.

**Methods:**

A retrospective cohort study was conducted on 940 SCT patients and 1263 matched non-SCT controls who underwent limb surgeries using tourniquets between 1978 and 2018. Patient data were gathered from hospital records, blood bank information, and postoperative haemoglobin electrophoresis. Outcomes assessed included the incidence of venous thromboembolism (VTE), pulmonary embolism (PE), phlebitis, peripheral nerve impairment, and superficial infection. Covariates included age, sex, ethnicity, and diabetes status.

**Results:**

Among SCT patients, 75% were unaware of their carrier status at surgery. VTE incidence was significantly higher in SCT patients (10%) than non-SCT controls (2%), especially after prolonged tourniquet use and in upper limb procedures without anticoagulation. PE occurred in 3% of SCT patients, versus 1% in controls. Diabetes, more prevalent in SCT individuals (9% vs. 6%), further increased the complication risk. Tourniquet inflation time did not differ significantly between groups; however, neurologic complications and phlebitis were more common in SCT patients, particularly those with Mediterranean ancestry. Notably, SCT carriers of African and Mediterranean descent experienced higher complication rates than non-carriers, though complications were not confined to any single ancestry.

**Conclusion:**

SCT is associated with an increased risk of thrombotic and neurologic complications during tourniquet, particularly in patients with undiagnosed SCT or diabetes and in procedures with extended ischaemia time. These results confirm the perception of SCT as a benign condition, but call for further clinical guidelines regarding tourniquet use in SCT carriers.

One ongoing debate in orthopaedic surgery concerns the use of tourniquets in patients with Sickle cell trait. Sickle cell trait (SCT), which is the heterozygous carrier form of sickle cell disease (SCD) anaemia, is estimated to affect approximately 300 million people worldwide [1]. SCT occurs when an individual carries one copy of the sickle haemoglobin (HbS) mutation. The HbS mutation arises from a single-nucleotide substitution in the gene that encodes the beta subunit of haemoglobin, specifically replacing thymine with adenine. This nucleotide change results in valine substituting for the amino acid glutamic acid.

The actual prevalence of sickle cell trait (SCT) may significantly exceed current estimates because many individuals remain unaware that they carry the trait [2], and some countries do not record SCT cases due to legal considerations [3]. The detection of SCT requires laboratory tests such as haemoglobin electrophoresis, chromatography, and DNA testing [2–3]. Individuals with SCT have both normal haemoglobin (HbA) and sickle hemoglobin (HbS) in their red blood cells (RBCs). Typically, HbA is predominant (> 50%), with HbS present in smaller amounts (approximately 40%), which limits HbS polymerization and the resulting sickling of RBCs in low oxygen conditions.

SCT prevalence is notably higher in regions with high malaria transmission, including West and Central Africa, the Mediterranean area (encompassing parts of Europe, North Africa, and the Middle East), and South Asia. This heightened prevalence (Fig. 1) is attributed to SCT’s protective effect against severe malaria. Although individuals with SCT are susceptible to parasitemia from Plasmodium falciparum infection, they experience significantly less severe clinical outcomes, with up to a 90% reduction in severe complications compared to individuals without SCT [4, 5].

**Fig. 1:**
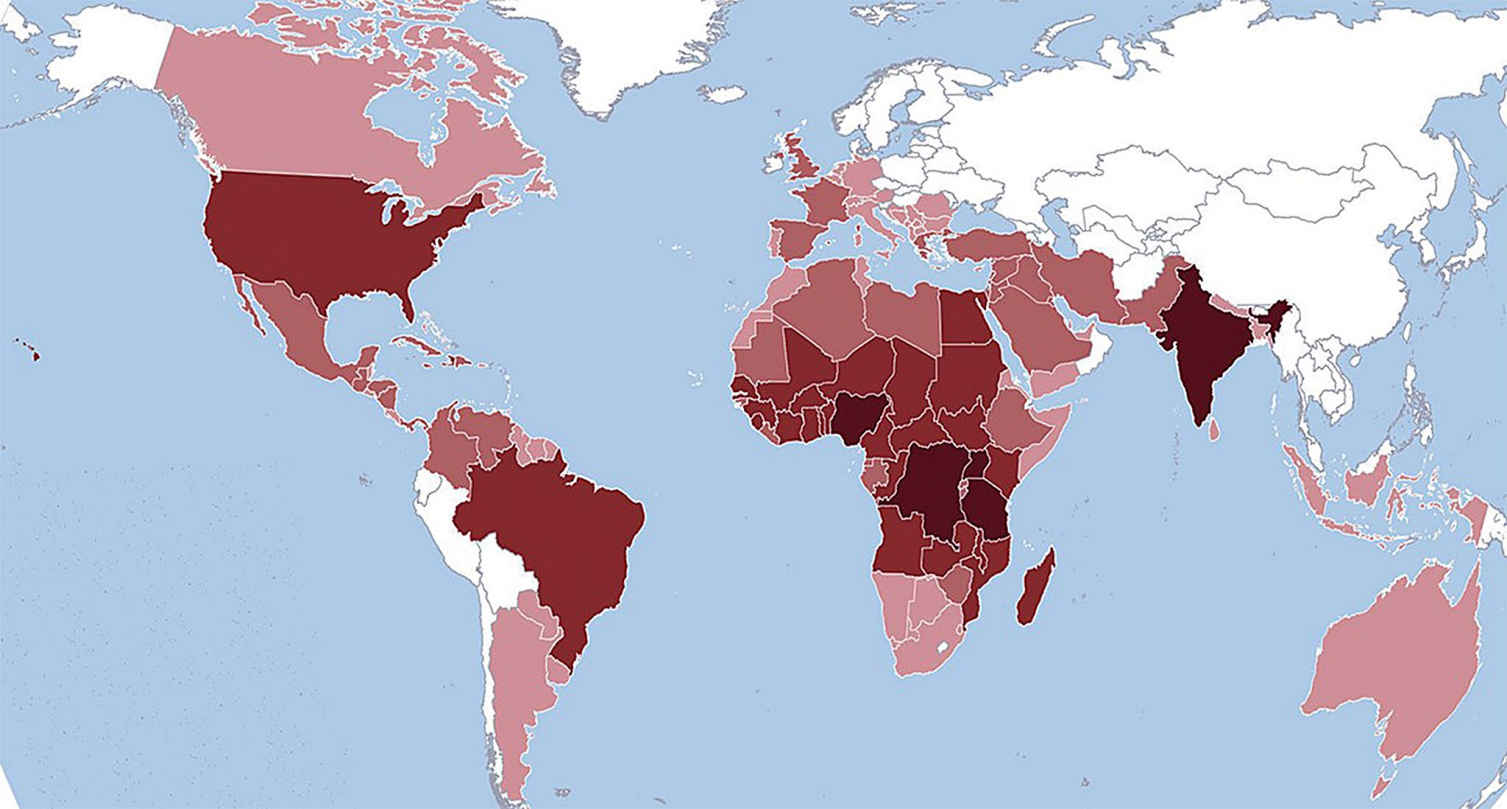
Sickle cell trait (SCT) prevalence in the world

In the Americas, SCT prevalence peaks among populations with ancestry linked to West and Central Africa. Globally, SCT is frequently misunderstood as affecting exclusively individuals identifying as Black or of African ancestry, despite its broader distribution across diverse populations. SCT is also found in the White population of the Mediterranean areas and in White emigrants from these Mediterranean areas in North and South America, but at a lower frequency. In Italy, Greece, Albania, and the south of France, HbS was historically almost exclusively present in regions with malaria. However, due to the migration flows, SCT is now present throughout the Italian, Greek, United Kingdom, and French national territories, as in emigrants from these areas from all over the world.

SCT is typically considered benign, with most carriers remaining asymptomatic throughout life. However, under extreme physiological stress—such as hypoxia, dehydration, or intense exertion—SCT has been associated [6, 7] with an increased risk of complications, including venous thromboembolism, exertional rhabdomyolysis, and vaso-occlusive events.

Tourniquets induce temporary, localized ischemia, which raises theoretical concerns about triggering red blood cell sickling and ischaemic injury in carriers of the HbS allele. Despite these concerns, there is limited empirical evidence guiding best practices, and the existing literature includes only a small number of documented cases involving surgical tourniquet use in SCT patients. For instance, Pignatti et al. [8] identified only 66 such cases reported in the literature worldwide.

Complicating this issue is the fact that many individuals (either white or black population) with SCT are unaware of their carrier status in the absence of Haemoglobin electrophoresis. This lack of awareness is especially critical in populations with higher SCT prevalence—such as individuals of African descent, where the carrier rate ranges from 15 to 20%. In such populations, the coexistence of other comorbidities, notably diabetes [9] with its associated microangiopathies, may further increase surgical risk when using a tourniquet. Unlike patients with diagnosed SCD anemia, who are typically identified early and managed with perioperative precautions and oxygen, undiagnosed SCT carriers may be inadvertently exposed to tourniquet surgical risks. Still, according to the outcomes of different studies and case reports, these risks are not uniform [8].

This study aims to clarify the clinical relevance of SCT in the surgical setting with a tourniquet and provide guidance regarding tourniquet use, particularly for populations with limited access to genetic screening.

## Materials and methods

This study used Hospital patient data, information from the blood bank, and a test before surgery to identify patients with an SCT. This study aimed to provide an appropriate contextualization of the association between SCT and tourniquet by evaluating the risk of complications among individuals with SCT, irrespective of race or ethnicity, and calculating comparative risks with patients without SCT.

### Patients

The data of a cohort of 940 SCT patients (Table 1) who underwent surgery between 1978 and 2018 and were performed with a tourniquet were reviewed. Individuals with Hb SS disease, Hb C, or β-thalassemia mutations were excluded and have been reported in another study [10].

Among these 940 SCT patients, we identified 120 procedures where the tourniquet time exceeded 120 min. The patients’ gender, age, and medical comorbidities, such as smoking and diabetes, were recorded. We collected procedure-specific data, including surgery details, tourniquet time, location, indications for both surgery and tourniquet use, and whether it involved the upper limb or lower limb.

**Table 1:** Participants

Group	Total Patients	Male / Female	Mean Age (years)	Age Range (years)	Lower Limb Cases	Upper Limb Cases
SCT Group	940	557 / 383	53	18–85	616	324
Non-SCT Group	1263	748 / 515	54	18–87	828	435

The study group (940 patients) was matched for comparison to a control group of non-SCT patients followed by the same team undergoing the same procedures in the same hospital during the same period (1978–2018). While matching for tourniquet time, lower limb and upper limb surgery, surgery and tourniquet location, the control (non-SCT) group consisted of 1263 matched non-SCT patients with 120 patients identified with a tourniquet time longer than 120 min. Patients were randomly selected from the matched dataset for the control group until a cohort of 120 individuals with a tourniquet time greater than 120 min was also obtained and matched for the same ancestry but without SCT.

#### Study group 940 sickle cell trait patients (SCT) with tourniquet

The patient cohort included 557 men and 383 women, totaling 940 individuals. The average age of the patients at the time of surgery was 53 years, with ages ranging from 18 to 85 years.

Surgery indications for the lower Limb (616 cases) included knee arthroplasty for osteoarthritis (207 cases) and rheumatoid arthritis (28 cases). Knee arthroscopy was performed in 70 patients. High tibial osteotomy was performed for knee osteoarthritis (187 cases). Ankle surgery was indicated for ankle fracture (64 cases) and hallux valgus (60 cases).

Upper Limb (324 cases): Indications included elbow fractures (68 cases), forearm fractures (98 cases), wrist fractures (30 cases), wrist arthroscopy (20 cases), and carpal tunnel syndrome (108 cases).

#### Control group (1263 patients)

The patient cohort consisted of 748 men and 515 women, totaling 1263 patients. The patients’ age at the time of surgery averaged 54 years, ranging from 18 to 87 years.

Patients in this study group were selected based on the following criteria:

Lower Limb (828 cases): Indications included total knee arthroplasty for knee osteoarthritis (278 cases) and rheumatoid arthritis (65 cases). High tibial osteotomy was performed for knee osteoarthritis (236 cases). Knee arthroscopy was performed in 42 knees. Ankle surgery was indicated for ankle fracture (99 cases) and hallux valgus (108 cases).

Upper Limb (435 cases): Indications included elbow fractures (91 cases), forearm fractures (132 cases), wrist fractures (47 cases), wrist arthroscopy (20 cases), and carpal tunnel syndrome (145 cases).

## Methods

According to the complications highlighted in the literature regarding tourniquet use in patients with Sickle Cell Disease anemia, this study focuses on the duration of the tourniquet and general complications. Painful sickle cell crises, phlebitis, and pulmonary embolism were evaluated, alongside skin complications and peripheral nerve impairment. Previous venous thromboembolism (VTE) and the outcome of a new VTE associated with surgery, as well as subsets of deep vein thrombosis (DVT) and pulmonary embolism (PE), were assessed through data analysis. The provoking factors of VTE were also evaluated, including category-based factors such as “another condition, such as cancer,” “medication,” “pregnancy,” “prolonged immobility,” “overweight,” or “surgery or injury.”

Covariates: Patient characteristics were collected to adjust for potential influences on adverse outcomes. These characteristics included age, sex, ethnicity, body mass index (BMI), smoking status, and diabetes. Covariates were selected to control for cohort heterogeneity and comorbidities, which may influence a surgeon’s decision to extend the tourniquet time. Type 2 diabetes was defined as the presence of any one of the following at the baseline examination: fasting glucose ≥ 7.0 mmol/l (126 mg/dl); non-fasting glucose ≥ 11.1 mmol/l (200 mg/dl); haemoglobin A1c ≥ 6.5%; current use of diabetic medication; or a positive response to the question, “Has a doctor ever told you that you had diabetes (sugar in the blood)?” Anticoagulation was recorded after surgery.

### Statistics

Descriptive statistics summarized the patients’ demographic properties. Chi-squared tests were employed to test for potential associations between tourniquet use and categorical variables (sex, race, ethnicity, and comorbidity). T-tests assessed differences in means for continuous variables (including age and operative time) based on tourniquet use.

## Results

### Participants

A total of 2203 patients (940 “SCT” and 1263 “non-SCT”) who underwent surgery with a tourniquet met the criteria for this analysis.

#### SCT group

The patient was unaware of their SCT carrier status at the time of surgery and tourniquet application in 703 (75%) of the 940 patients with the trait. This status was determined using information from the blood bank and a test conducted after surgery. For the remaining patients, the status was derived from tests (haemoglobin electrophoresis) performed upon suspicion of sickle cell disease in the family or other haemoglobinopathies. The prevalence of SCT varied significantly among genetic ancestry groups within the 940 patients, with the highest frequency observed among Black individuals of West and Central African ancestry (650 out of 940 patients (69%), 304 of whom had known status), followed by South Mediterranean Europe (129 patients (14%), all with unknown status), North African Mediterranean individuals (93 patients (10%), 19 with known status), South Asian (Indian, Pakistani, Malaysian, etc.) ancestry (62 patients (6%), all with unknown status), and the lowest frequency among individuals of North Western European ancestry (6 patients (1%), all with unknown status). Diabetes type 2 was present in 7% of patients, but for 43% of patients, it was unknown at the time of surgery.

#### Non-SCT group

The non-SCT carrier status was identified in 1263 patients during surgery or afterward. This status was obtained from information provided by the blood bank. Among genetic ancestry groups, the prevalence of non-SCT status was comparable to that of the SCT group, except for individuals of northwestern European ancestry, where the number was voluntarily increased to establish a comparison in the non-SCT control group: Non-SCT Black individuals of West and Central African ancestry (663 patients), followed by Non-SCT South European Mediterranean individuals (138 patients), Non-SCT North African Mediterranean individuals (112 patients), South Asian individuals from India, Pakistan, Malaysia, etc. (83 patients), and individuals of northwestern European ancestry (260 patients).

#### Comorbidities in the two groups

Patients with SCT were of the same age but tended to have more comorbidities, particularly diabetes and a prior history of VTE, compared to those without the SCT trait. The groups did not differ in BMI or in the percentage of patients taking pain medication preoperatively. Among the individuals with SCT, 28 (3%) reported a history of DTE, compared to one patient (12%) without SCT. A history of PE was identified in two patients (20%) with SCT compared to 0.5 patients (5%) without SCT. Type 2 diabetes was present in 9% of patients in the SCT group and in 6% of the non-SCT group; however, the diabetes status was unknown for 43% of patients at the time of surgery. In the SCT group, Type 2 diabetes was approximately twice as prevalent in African Ancestry (11%) as in South European Mediterranean (6%), and also more frequent in the SCT group as compared with the Non-SCT group. Among the patients with SCT, one case of chronic kidney disease (CKD) was noted, along with two instances of hip osteonecrosis related to corticosteroid use.

### Tourniquet

There was no difference in operative time between the groups. The mean tourniquet inflation times were 66 min (range 30 to 203 min) and 67 min (range 35 to 236 min) in the SCT group and the group without SCT, respectively. All 57 procedures with tourniquet inflation times longer than 90 min were complex surgeries, with 52 of these being revision procedures. The patients received general anaesthesia, often supplemented by a postoperative nerve block. The average uninterrupted tourniquet duration for these 57 procedures was 191 min, comprising 30 in the SCT group and 27 in the non-SCT group. The 57 cases with the longest tourniquet times included interruptions during inflation to allow for reperfusion. For these cases, the first tourniquet inflating session lasted between 58 and 75 min, while the second session ranged from 187 to 196 min. Therefore, all patients had an uninterrupted tourniquet time exceeding 180 min. Tourniquet pressure was 300 mm Hg for all patients. Emergency surgery was performed for fractures in 15% of patients in the SCT group and 17% in the non-SCT group. All patients who had abnormal tourniquet times were either patients who were unknown to have SCT for non-emergency, or who were unknown to have diabetes while undergoing emergency surgery.

### Complications (Table 2)

**Table 2:** Complications of tourniquet use in sickle cell trait (SCT)

**VTE and PE Incidence by Group**						
**Group**	**VTE**	**(%)**	**PE**	**(%)**		
SCT (*n* = 940)	Nb 97	10%	Nb 31	3%		
Non-SCT (*n* = 1263)	NB 25	2%	NB 11	1%		
**VTE Incidence in lower limb with Tourniquet Time > 120 min**						
**Group**	**Cases (n)**		**VTE Cases**			
SCT	30		9			
Non-SCT	27		0			
**Neurologic Complications Post-Surgery**						
**Condition**	**SCT Patients **(***n*** = 940)		**Comments**			
Transient sensory loss	112		Recovered in 2 weeks; all diabetic or long tourniquet use			
Diabetes at surgery	67 of 112		unknown status at surgery			
SCT at surgery	78 of 112		Unknown status at surgery			
**VTE by Ancestry in SCT Group**						
**Ancestry**	**SCT Patients (n)**		**VTE Cases**		**%**	
Black African	650		59		9%	
South European Mediterranean	129		21		16%	
North African Mediterranean	93		10		11%	
South Asian	62		1			
North Western European	6		0			

#### VTE and pulmonary embolism

A new history of DTE after surgery was reported by 97 (10%) individuals with SCT, compared to 25 (2%) without SCT. A new history of PE was found in 31 (3%) individuals with SCT, compared to 11 (1%) without SCT. Additionally, a more significant number of individuals without SCT (18 cases) reported a provoked VTE compared to those with SCT (8 cases). Even after excluding the 26 instances of provoked VTE, we identified a significant difference in the number of individuals with VTE between those with SCT (89 cases among 940) and those without SCT (7 cases among 1263), particularly for upper limb surgery (25 SCT cases among 324 cases without anticoagulation), and for lower limbs when the tourniquet time was prolonged (9 cases despite anticoagulation among 30 cases with more than 120 min tourniquet).

Considering genetic ancestry, among SCT carriers, the highest frequency of VTE events occurred in Mediterranean individuals (21 cases among 129 South European Mediterranean individuals and ten among 112 North African Mediterranean individuals, followed by Black Africans (59 cases among 650 cases). We also investigated whether other demographic factors and comorbidities influenced the VTE positivity of SCT carriers. Most of these variables were not significantly linked to VTE positivity, possibly due to the small sample size. However, diabetes was more prevalent in the 29 SCT carriers (5 type 2 diabetes) who tested positive for VTE than in those (8% diabetes) who tested negative, particularly among individuals of Black African ancestry.

#### Other complications

Twelve patients developed a superficial infection that resolved with antibiotics. None of the patients reported tourniquet-related pain in the thigh or arm. A total of 138 patients had complications recorded in addition to VTE. One hundred twelve patients experienced a transient loss of sensation in their legs, which fully recovered within two weeks. All these patients were either diabetic (112 individuals) or had a history of long-term tourniquet use (26 individuals), and 78 were unknown SCT patients at the time of surgery. We investigated whether demographic factors and comorbidities (such as diabetes) influenced the neurologic complications. The diabetes status was also unknown for 67 of these 112 individuals at the time of surgery.

## Discussion

Sickle cell trait (SCT) has traditionally been considered a benign carrier state of the sickle haemoglobin (HbS) gene. However, emerging evidence has suggested an association between SCT and several medical complications, particularly under extreme physiological conditions. This section comprehensively analyzes tourniquet complications observed in SCT individuals, highlighting these conditions’ prevalence and clinical significance.

Although pneumatic tourniquets are common in orthopaedic surgery, our understanding of safe application times is still incomplete. Most clinical evidence focuses on knee surgery and the white population. It is essential to review evidence defining safe time limits and to examine the outcomes of patients with prolonged tourniquet durations and the different ancestral populations involved. How can we assess surgical tourniquet risks in patients with sickle cell trait when 75% are unaware they have it and may also be unknowingly diabetic? One might suggest a simple solution: always perform surgery without a tourniquet! However, as Sterling Bunnell, the founding father of hand surgery, stated [11]: “Operating on a hand without a tourniquet is like trying to fix a watch in a bottle of ink, “and the same principle can apply to articular fractures of the lower limb! Acknowledging the potential for serious tourniquet complications, orthopaedic surgeons strive to adhere to the accepted safe tourniquet times (less than one hour for the upper limb and less than 90 min for the lower limb). Nevertheless, several factors can lead to exceeding these limits: complex surgical procedures and the possibility of intraoperative complications are scenarios where this may occur. Given the notion that complications may arise in patients with sickle cell trait (SCT) due to prolonged tourniquet time, we retrospectively analyzed our patients’ data to determine whether there was a higher incidence of complications in surgeries involving tourniquets, particularly with tourniquet durations of two hours or more in patients with SCT compared to those without it.

Historically, SCT was viewed as a clinically insignificant genetic condition [4–12]. However, since the mid-20th century, sporadic reports have challenged this view [13]. Studies involving military recruits, athletes, and laborers have linked SCT to adverse events during exertion. Over time, large-scale epidemiological studies have demonstrated associations between SCT and chronic kidney disease, as well as a significantly elevated risk of exercise-related sudden death [1, 14, 15]. Still, the results have been inconsistent, and consensus remains elusive. Moreover, although the vast majority of SCT carriers will never experience an exertion-related event, large epidemiological studies [1] have reported a higher risk of exercise-related sudden death in SCT carriers compared to non-SCT individuals.

In medical literature, SCT is often categorized as a “Black” trait. This classification partly arises from research evaluating statistical variations in prevalence rates across different racial and genetic ancestry groups. Our research demonstrates that SCT is prevalent among various ancestry groups and shows a consistent VTE risk pattern independent of genetic ancestry factors. However, in our study, the risk of phlebitis is higher in the White South European Mediterranean population than in the Black population carrying the SCT trait, not only for lower limb but also for upper limb surgery. We also observe that the risk for SCT patients of developing phlebitis is greater than that of patients who are homozygous for sickle cell disease [10], likely because the populations are of different ages and the homozygous sickle cell population is more closely monitored medically, particularly concerning oxygen.

Studies on tourniquet use in limb surgery for patients with sickle-cell trait are inconsistent. For instance, Pignatti et al. [8] identified only 66 cases of SCT reported in the literature worldwide. Interventions varied significantly regarding tourniquet type, operative procedures, and perioperative care. Most studies combined SCT and sickle-cell anaemia patients [16–23]. In our series, we did not observe any sickle cell crisis. We found some sickled red cells in bone cuts of patients undergoing knee arthroplasty, but this does not imply that sickling was present in the vascularized bone. A distinct advantage of this study was the identification of SCT carriers within the population. This is linked to our hospital being a national monitoring center for sickle cell patients for half a century [10, 24]. This discovery carries two critical implications. Firstly, many SCT carriers might be unrecognized or misclassified. Secondly, and possibly more crucially, our data show that the SCT carrier rate is significantly higher than prior estimates, and most SCT carriers in the general population are unaware of their status and diabetes. As a result, many SCT carriers miss the opportunity for additional care. One limitation of this study was its relatively small sample size. Nevertheless, this study represents one of the most extensive investigations regarding the association between tourniquet use and SCT.

In conclusion, sickle cell trait is one of the most common hemoglobin carrier states worldwide and concerns many families with patients having bone pathologies related to the disease in the world [25–27]. Although SCT typically allows carriers to maintain a normal life expectancy and quality of life, it can be associated with some complications under tourniquet conditions, such as thrombophlebitis. Therefore, identifying patients’ SCT carrier status is beneficial, primarily due to the positive effects this information may have on their health. Furthermore, increasing awareness of these clinical manifestations and their prevention and management can serve as a valuable resource for all healthcare providers involved in this issue.

## Data Availability

No datasets were generated or analysed during the current study.
